# Serum Adalimumab Levels After Induction Are Associated With Long-Term Remission in Children With Inflammatory Bowel Disease

**DOI:** 10.3389/fped.2021.646671

**Published:** 2021-05-04

**Authors:** Marianna Lucafò, Debora Curci, Matteo Bramuzzo, Patrizia Alvisi, Stefano Martelossi, Tania Silvestri, Veronica Guastalla, Flavio Labriola, Gabriele Stocco, Giuliana Decorti

**Affiliations:** ^1^Advanced Translational Diagnostics Laboratory, Institute for Maternal and Child Health Scientific Institute for Research, Hospitalization and Healthcare (IRCCS) Burlo Garofolo, Trieste, Italy; ^2^Reproductive and Developmental Sciences, University of Trieste, Trieste, Italy; ^3^Gastroenterology, Digestive Endoscopy, and Nutrition Unit, Institute for Maternal and Child Health-Scientific Institute for Research, Hospitalization and Healthcare (IRCCS) “Burlo Garofolo”, Trieste, Italy; ^4^Pediatric Gastroenterology Unit, Maggiore Hospital, Local health Centre (AUSL) Bologna, Bologna, Italy; ^5^Pediatric Gastroenterology Unit, Cà Foncello Hospital, Treviso, Italy; ^6^Single metropolitan laboratory (LUM) Autoimmunity and Allergy, Local Health Centre (AUSL) Bologna, Bologna, Italy; ^7^Department of Medicine, Surgery, and Health Sciences, University of Trieste, Trieste, Italy; ^8^Department of Life Sciences, University of Trieste, Trieste, Italy

**Keywords:** inflammatory bowel disease, children, adalimumab, drug levels, anti-TNF, therapeutic drug monitoring

## Abstract

**Introduction:** Adalimumab is effective in inducing and maintaining remission in children with inflammatory bowel diseases (IBD). Therapeutic drug monitoring is an important strategy to maximize the response rates, but data on the association of serum adalimumab levels are lacking. This study aimed to assess the association of adalimumab concentrations at the end of induction and early during maintenance for long-term response.

**Materials and Methods:** Serum samples for adalimumab level measurement were collected during routine visits between adalimumab administrations and therefore not necessarily at trough, both during the induction (week 4 ± 4) and maintenance phases (week 22 ± 4, 52 ± 4, and 82 ± 4). Adalimumab and anti-adalimumab antibodies were measured retrospectively using enzyme-linked immunosorbent assays (ELISA). Disease activity was determined by Pediatric Crohn's Disease Activity Index or Pediatric Ulcerative Colitis Activity Index.

**Results:** Thirty-two children (median age 14.9 years) were enrolled. Sixteen, 15, 14, and 12 patients were in remission at weeks 4, 22, 52, and 82, respectively. Median adalimumab concentration was higher at all time points in patients achieving sustained clinical remission. Adalimumab levels correlated with clinical and biochemical variables. Adalimumab concentration above 13.85 and 7.54 μg/ml at weeks 4 and 22 was associated with remission at weeks 52 and 82.

**Conclusions:** Adalimumab non-trough levels are associated with long-term response in pediatric patients with IBD.

## Introduction

Pediatric inflammatory bowel disease (IBD), encompassing Crohn's disease (CD) and ulcerative colitis (UC), accounts for about 25% of all cases and a more complicated and aggressive course than adult-onset IBD, requiring appropriate treatment escalation, has been reported. Monoclonal antibodies targeting TNF-alpha play a pivotal role in the treatment of IBD, in particular, in patients with severe disease activity and refractoriness to standard therapies (1, 2). Adalimumab, a fully humanized monoclonal immunoglobulin G1 administered subcutaneously, is effective in inducing and maintaining remission in children with CD and is used off-label as secondary-line therapy in UC. Pediatric studies, which considered both anti-TNF therapy-naïve patients and patients receiving adalimumab after infliximab failure, showed remission rates after 12 months of therapy of 41 and 44% for CD and UC, respectively. However, 12–15% of the children with IBD who initially respond to therapy relapse within a year, requiring additional treatments (1, 2). Therapeutic drug monitoring (TDM) has been used to modulate drug concentrations and improve clinical response (3). TDM could be performed after the loss of response (reactive TDM) or during clinical remission to reduce the risk of treatment failure in the long-term period (proactive TDM) (4). While the usefulness of the reactive strategy has been described, proactive TDM still requires proof of effectiveness (5).

In adult patients with CD and UC, the cutoff of 5 μg/ml for adalimumab trough concentration was found to be associated with clinical remission (6, 7), while higher serum adalimumab concentrations (8–12 μg/ml) were needed when mucosal healing was the evaluated outcome (8). On the other hand, lower adalimumab concentrations correlated with the risk of anti-adalimumab antibody (ADA) development (9).

To support the decision-making strategy for optimizing treatment, some studies also evaluated the correlation of the trough levels at optimal points with the long-term disease activity. For infliximab, levels >3.5 μg/ml at the end of induction have been shown to predict remission at 52 weeks both in children and adults (10–13). Similarly, a cutoff of 5 μg/ml for adalimumab levels was identified as a good predictor for clinical response at 52 weeks (13). To date, no data regarding the correlation between adalimumab levels at the end of induction and long-term outcomes are available.

The primary aim of this study was to evaluate the association between adalimumab concentration and disease activity in a cohort of pediatric IBD patients. The clinical disease activity was assessed at defined time points (at the end of induction, at week 22, 52, and 82). The secondary aims were to evaluate the association of adalimumab levels at the end of the induction and early during maintenance with clinical response and remission at 52 and 82 weeks and the correlation between adalimumab levels and the development of ADAs.

## Materials and Methods

### Patients

This retrospective cross-sectional study considered patients with a diagnosis of CD or UC according to the Porto criteria (14), aged between 6 and 18 years and treated with adalimumab, enrolled at three Italian pediatric facilities (Gastroenterology, Digestive Endoscopy and Nutrition Unit, Institute for Maternal and Child Health-IRCCS “Burlo Garofolo,” Trieste; Pediatric Gastroenterology Unit, Maggiore Hospital, AUSL Bologna; Cà Foncello Hospital, Treviso) between January 2013 and September 2019. Adalimumab was started in case of treatment failure or intolerance to standard therapies (steroids, exclusive enteral nutrition and immunomodulators) or infliximab. In selected patients, adalimumab was used as first-line treatment (15). Concomitant therapy with immunomodulators was permitted. Exclusion criteria were disease needing urgent surgery, presence of an ileostomy or colostomy, infectious complications, or other contemporary uncontrolled medical conditions. Children were treated according to a therapeutic protocol consisting of subcutaneous administration of adalimumab on day 1 at 160 and 80 mg, and on day 15 at 80 and 40 mg, for body weight ≥40 or <40 kg, respectively (induction phase), followed by every other week at 40 or 20 mg for body weight ≥40 or <40 kg, respectively (maintenance phase). In case of clinical evidence of loss of response, therapy with adalimumab could be escalated by shortening intervals between injections to once a week.

Demographic and clinical data, including age at diagnosis, localization and behavior of the disease according to the Paris classification (16), and therapies previous to adalimumab, were collected. Details on adalimumab treatment included age at adalimumab start, initial dose and concomitant therapies, and need for interval shortening during maintenance. Biochemical tests during adalimumab treatment were recorded and included C reactive protein (CRP), albumin, and fecal calprotectin. Serum samples for adalimumab level measurement were collected during routine visits between adalimumab administrations and therefore not necessarily at trough, both during the induction (week 4 ± 4) and maintenance phases (week 22 ± 4, week 52 ± 4, and week 82 ± 4). Samples were stored at −20°C and were analyzed retrospectively. All patients with complete clinical response (disease activity score ≤10) to treatment after induction continued therapy with adalimumab and were followed until week 82.

### Clinical Remission

Clinical disease activity was assessed using pediatric Crohn's disease activity index (PCDAI) and pediatric ulcerative colitis activity index (PUCAI) for CD (17) and UC patients (18), respectively, at baseline, at the end of induction (4 weeks), and during maintenance phase at 22, 52, and 82 weeks. The disease was defined in remission if the disease activity index was ≤10. Loss of response was considered either as clinical worsening (PCDAI/PUCAI > 10) in a patient who had previously attained clinical response or as the need for treatment intensification.

### ELISA Assay: Measurement of Adalimumab and ADA Levels

Adalimumab levels were measured using the commercial Promonitor ELISA assay (Proteomika S.L., subsidiary of Progenika Biopharma S.A., Spain) on sera from peripheral blood samples collected at the time points specified above. An ELISA kit was used according to the manufacturers' instructions, and the lower and upper limits of quantification were 0.024 and 12 μg/ml, respectively. For sera above the limit of the kit (12 μg/ml), a further dilution was required. ADAs were measured when adalimumab serum levels were <1.5 μg/ml (Promonitor Anti-Adalimumab ELISA assay; Proteomika S.L., subsidiary of Progenika Biopharma S.A., Spain). ADA levels higher than 10 arbitrary units per milliliter (AU/ml) were considered positive.

### Statistical Analysis

Statistical analysis was performed using the R software (version 3.4.2). The association between adalimumab concentrations and the therapeutic response was evaluated in a univariate analysis by linear mixed effect (LME) models, which allow to account for repeated measures, using patient's response to adalimumab as the dependent variable and adalimumab serum levels as the independent variable. To find the optimal drug levels for predicting efficacy, the association between adalimumab concentrations and response at the various time points was identified using logistic regression analysis. The potential confounding effect of adalimumab dose was tested by considering the difference in adalimumab weight normalized dose at different time points by LME logistic regression analysis. Receiver operating characteristic (ROC) curves were then constructed for adalimumab concentrations, to determine the optimal cutoff, using the Youden index to predict clinical response. Sensitivity, specificity, and positive and negative predictive values of the cutoff point were analyzed. An analysis of the association between adalimumab concentrations considering all time points and the clinical and biochemical parameters [clinical scores, CRP, and fecal calprotectin] was carried out by the non-parametric Spearman's test. The potential effect of demographic and clinical patients' characteristics on clinical response was tested by LME analysis. For the clinical laboratory parameters, the normality of the distribution was evaluated by Shapiro's test. The association between ADAs and drug concentrations was determined by Spearman's test. Continuous data are presented as medians with interquartile ranges (IQRs) (represented as the interval between the first and the third quartile), and categorical data are presented as absolute numbers and percentages. *P* < 0.05 was considered statistically significant.

## Results

### Clinical Remission

Thirty-two pediatric patients with IBD were enrolled. The demographic characteristics of the population are summarized in Table 1. Twenty-eight patients (87.5%) had stopped infliximab before adalimumab, and 4 patients (12.5%) were anti-TNF naive. Infliximab was stopped because of adverse events in 13 patients (46.4%), loss of response due to anti-infliximab antibodies in 8 patients (28.6%), and loss of response during induction phase in 7 patients (25%). At adalimumab start, 8 patients (28.6%) were in clinical remission according to the PCDAI/PUCAI activity score, but had biochemical alterations (elevated CRP and fecal calprotectin levels); however, the pre-adalimumab therapy score was not associated with response or adalimumab concentration at later time points (pre-adalimumab therapy score vs. clinical response at 4, 22, 52, and 82 weeks: *P* = 0.47, *P* = 0.11, *P* = 0.56, and *P* = 0.67, respectively; pre-adalimumab therapy score vs. adalimumab concentration at 4 and 22 weeks: *P* = 0.88 and *P* = 0.86, respectively). Sixteen, 15, 14, and 12 patients were in remission at weeks 4, 22, 52, and 82, respectively. All patients were steroid free. Five (15.6%) patients lost the initial clinical response: 2 recovered spontaneously, while 3 shortened intervals between injections without the need for additional treatments (Supplementary Figure 1). Three patients (9.4%) were lost at follow-up before week 52 and 7 patients (21.9%) before week 82. No patient discontinued the treatment because of adverse event (anaphylactoid reaction). No statistical association between clinical response and demographic (age and gender) and clinical (type of IBD) characteristics of the population was found after logistic regression analysis (*P* > 0.05).

**Table 1:** Demographic and clinical characteristics of the population enrolled.

	**Overall (*n* = 32)**	**Crohn's disease (*n* = 24)**	**Ulcerative colitis (*n* = 8)**
Age-years (IQR)	14.88 [12.1–16.7]	14.72 [11.2–16.3]	16.46 [14.3–17.3]
Disease duration-months (IQR)	41.73 [23.3–67.4]	46.30 [31.2–68.8]	23.3 [19.9–30.9]
**Gender-*****n*** **(%)**			
Female	12 (37.5)	7 (29.2)	5 (62.5)
Male	20 (62.5)	17 (70.8)	3 (37.5)
**Disease activity index at inclusion-*****n*** **(IQR)**			
PCDAI		15 (8–20)	-
PUCAI		-	15 (8–20)
**Concomitant therapy-*****n*** **(%)**			
Steroids	-	-	-
Methotrexate	4 (12.5)	4 (16.7)	-
Azathioprine	-	-	-
Thalidomide	1 (3.1)	1 (4.2)	-
**Disease location-*****n*** **(%)**			
L1		1 (4.2)	-
L2		2 (8.3)	-
L3		18 (75)	-
L4		3 (12.5)	-
**Disease extent-*****n*** **(%)**			
E1		-	-
E2		-	-
E3		-	-
E4		-	8 (100)
**Disease behavior-*****n*** **(%)**			
B1		23 (95.8)	-
B2		1 (4.2)	-
B3		-	-
P		4 (16.7)	-
**Disease behavior-*****n*** **(%) Montreal classification**			
B1		23 (95.8)	-
B2		1 (4.2)	-
B3		-	-
**CD perianal fistulizing status:**			
YES-*n* (%)		4 (16.7)	-
NO-*n* (%)		20 (83.3)	-

*IQR, interquartile range; PCDAI, Pediatric Crohn's disease activity index; PUCAI, Ulcerative Colitis Activity index. Disease location according to the Paris Classification for Crohn's disease-L1, distal 1/3 ileum ± limited cecal disease; L2, colonic; L3, ileocolonic; L4, upper disease and disease behavior; B1, non-structuring; B2, structuring; B3, penetrating; P, perianal disease modifier; disease extent for ulcerative colitis-E1, proctitis; E2, left-sided disease; E3, extensive disease; E4, pancolitis (15)*.

### Adalimumab Concentrations and Disease Activity

**Figure 1:**
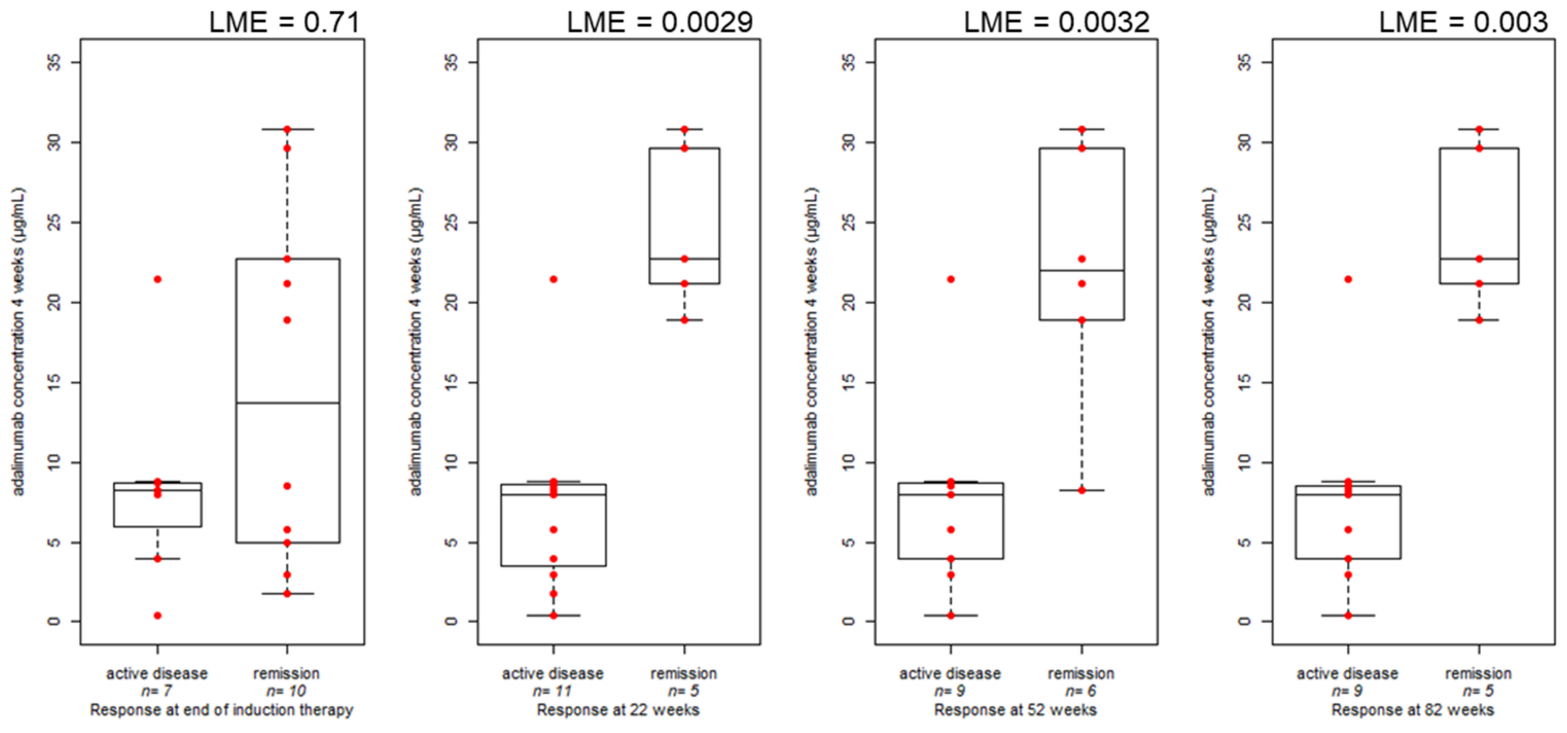
Boxplot comparing clinical disease activity at the end of induction (17 measurements in 13 patients), at 22 (16 measurements in 12 patients), 52 (15 measurements in 11 patients), and 82 weeks (14 measurements in 10 patients) and serum adalimumab concentration during induction therapy (4 weeks). The bold horizontal line represents the median value. *P*-values are from linear mixed effect model (LME), accounting for repeated measures.

Serum adalimumab levels at 4 weeks were correlated with clinical remission during maintenance. Higher serum adalimumab levels at week 4 were found for patients in clinical remission compared with patients with clinically active disease at week 22 (22.72 μg/ml, IQR: 8.4 vs. 8 μg/ml, IQR: 5.08 μg/ml, *P* = 0.0029), at week 52 (22 μg/ml, IQR: 8.4 vs. 8 μg/ml, IQR: 4.66 μg/ml, *P* = 0.003), and at week 82 (22.72 μg/ml, IQR: 8.39 vs. 8 μg/ml, IQR: 4.5 μg/ml, *P* = 0.003) (Figure 1).

**Figure 2:**
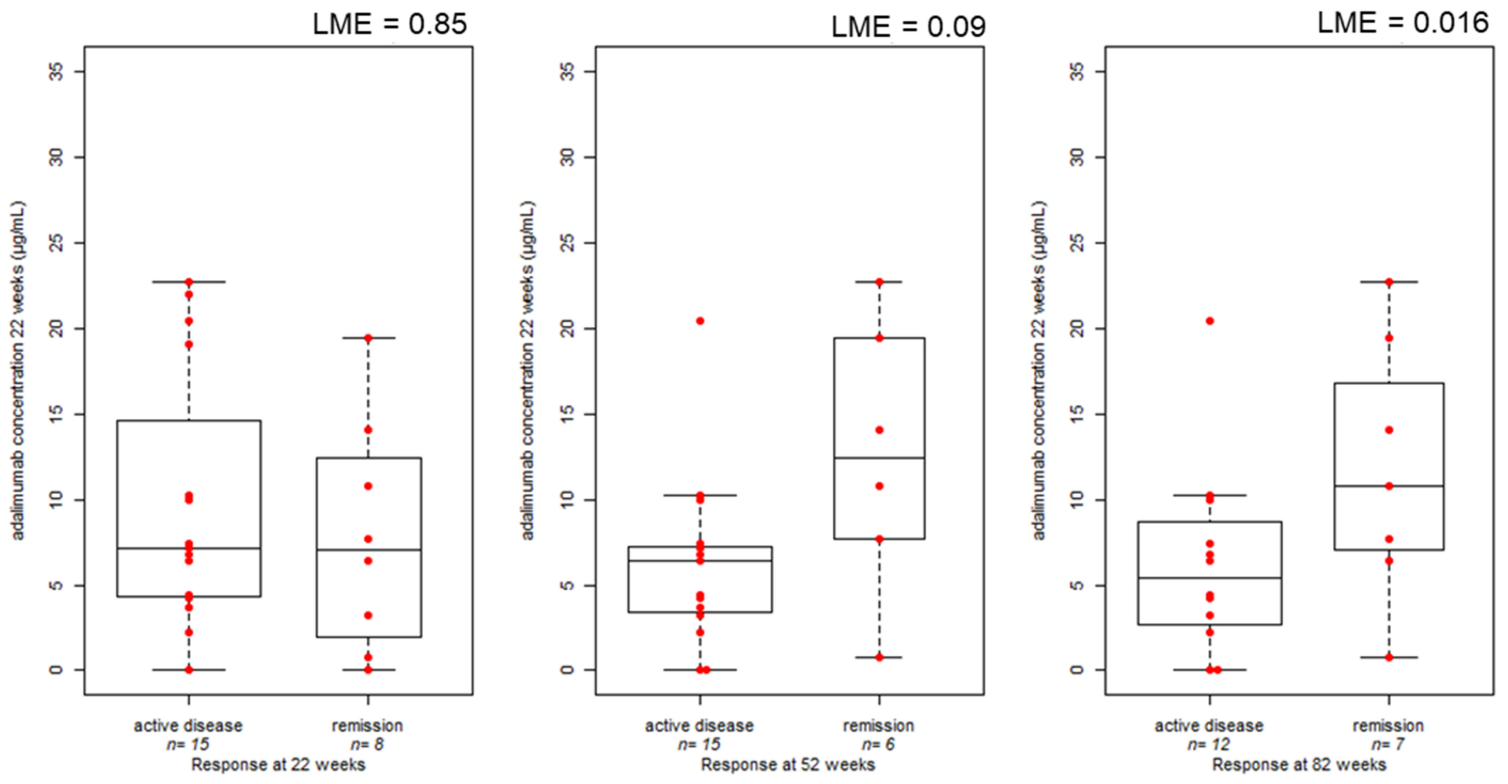
Boxplot comparing clinical disease activity at 22 (23 measurements in 19 patients), 52 (21 measurements in 18 patients), and 82 (19 measurements in 16 patients) weeks of treatment and serum adalimumab concentration during week 22. The bold horizontal line represents the median value. *P*-values are from linear mixed effect model (LME), accounting for repeated measures.

The same trend was observed also considering shorter timeframes in the induction interval and long term response (Supplementary Figure 2). However, median adalimumab levels measured at week 4 did not differ between patients in clinical remission and patients with clinically active disease at the end of induction (13.7 μg/ml, IQR: 17.13 vs. 8.26 μg/ml, IQR: 2.73 μg/ml, *P* = 0.71), and the same trend was observed considering median adalimumab levels at week 22 and the clinical response at week 22 (7.04 μg/ml, IQR: 9.6 vs. 7.14 μg/ml, IQR: 10.34 μg/ml, *P* = 0.85). Patients in remission at weeks 52 and 82 had higher adalimumab levels at week 22 compared with patients with clinically active disease (12.44 μg/ml, IQR: 9.62 vs. 6.38 μg/ml, IQR: 3.82 μg/ml, *P* = 0.09; and 10.8 μg/ml, IQR: 9.72 vs. 5.4 μg/ml, IQR: 5.09 μg/ml, *P* = 0.016, respectively) (Figure 2). Quartile analysis comparing the lowest vs. highest quartile exposure (Q1 vs. Q4) resulted in higher percentage of responder in Q4 at the different time point (Supplementary Figure 3). Patients' enrolment was done cross-sectionally and only for 7 patients; measurements were obtained both at 4 and 22 weeks.

**Figure 3:**
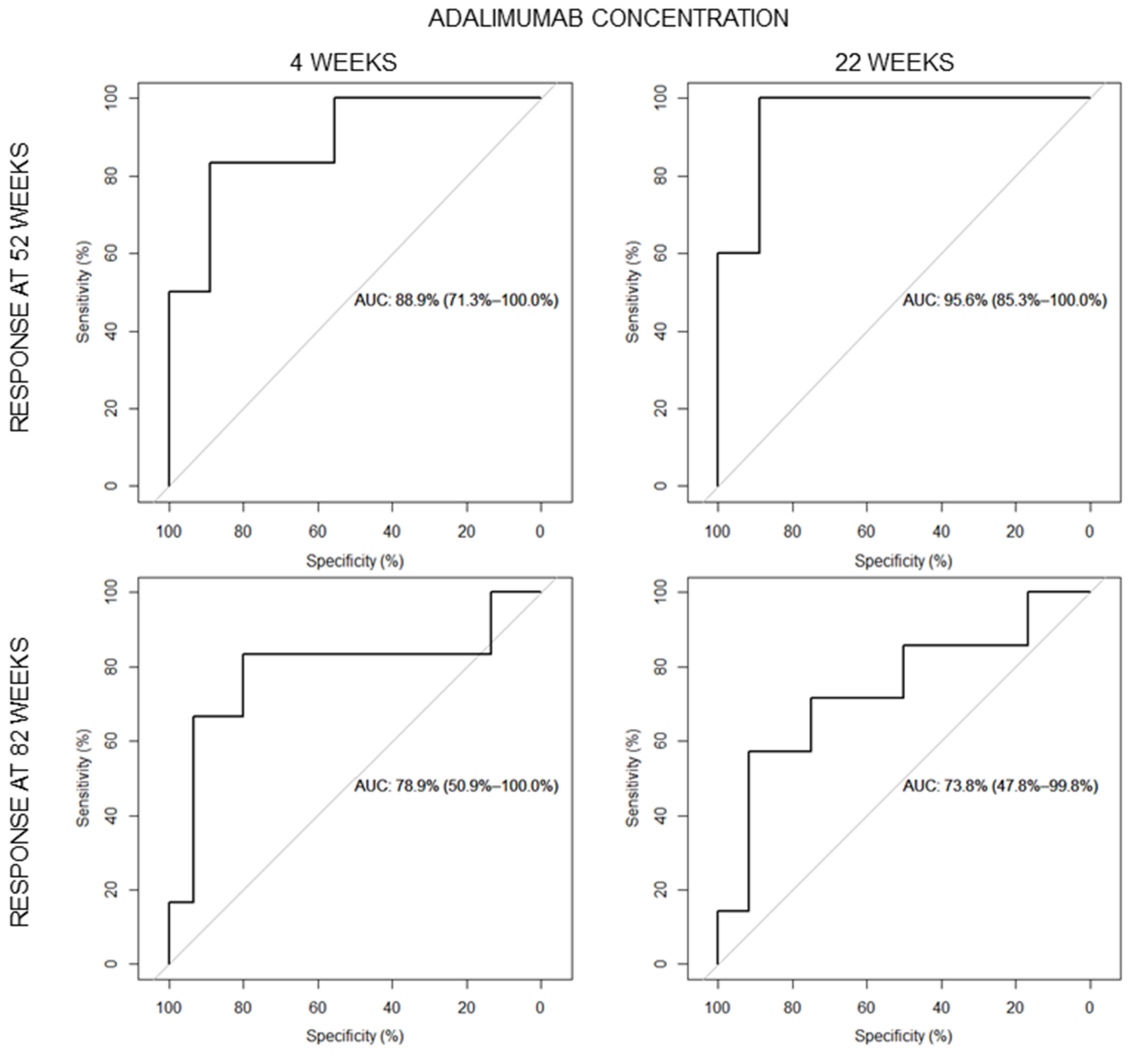
Areas under the receiver operating characteristic (ROC) curves for the serum adalimumab quantification at the end of induction and week 22 for clinical disease activity at 52 (above) and 82 weeks (below).

No significant difference in adalimumab dose, normalized on patients' weight, was observed at week 4, while at week 22, patients with clinically active disease were treated with higher adalimumab weight normalized doses (Supplementary Figure 4). ROC curves were constructed to assign optimal cutoff values for adalimumab levels at the end of induction therapy and after early maintenance phase to predict clinical response at 52 and 82 weeks (Figure 3).

Optimal cutoff values were 13.85 and 7.54 μg/ml at weeks 4 and 22, respectively, to predict clinical response at week 52. The cutoff values of 13.85 and 10.51 μg/ml were obtained at weeks 4 and 22, respectively, to predict clinical response at week 82. Logistic regression analysis confirmed that patients who reached the cutoff point of 13.85 μg/ml at the end of induction (6 patients, 5 in sustained remission both at weeks 52 and 82) had a higher probability of maintaining remission at weeks 52 and 82 compared with those who did not (nine patients, only one in sustained remission at week 52; eight patients, none in remission at week 82), with an odds ratio (OR) of 40.0 [95% confidence interval (CI) 2.0–794.3, *P* = 0.015] and OR of 62.33 (95% CI 2.13–1822.7, *P* = 0.016), respectively, at weeks 52 and 82. Patients above the cutoff point of 7.54 μg/ml during week 22 (8 patients, 5 reached remission at week 52) had a higher probability of maintaining remission at week 52 compared with those who did not (13 patients, only one in sustained remission at week 52) with an OR of 20 (95% CI 1.65–241.7, *P* = 0.018). Patients with a cut-off point of 10.51 μg/ml during week 22 reached remission at 82 weeks (5 patients, 4 in sustained remission) compared with those who did not (14 patients, only 3 in sustained remission) with an OR of 14.66 (95% CI 1.16–185.24, *P* = 0.037) (Supplementary Table 1).

The area under the ROC curve (AUC) was 88.9% at week 4 and 78.9% at week 22, considering the response at week 52, and 95.6 and 73.8%, respectively, at weeks 4 and 22, considering the response at week 82. The test had a sensitivity of 83.3% and specificity of 88.8% considering week 4 [positive predictive value (PPV) 83.3%; negative predictive value, NPV 88.8%] and a sensitivity of 83.3% and specificity of 80% at week 22 (PPV 62.5%; NPV 92.3%) for obtaining sustained remission at week 52. A sensitivity of 100% and a specificity of 88.9% were obtained considering week 4 (PPV 83.3%; NPV 100%) and a sensitivity of 57.14% and a specificity of 91.66% considering week 22 (PPV 80%; NPV 78.57%) for obtaining sustained remission at week 82.

### Correlation Between Serum Adalimumab Levels and Clinical and Biochemical Variables

Sex, age, and IBD type were not significantly associated with adalimumab concentration. The cause of infliximab failure and the concomitant treatment with immunomodulators did not affect adalimumab levels (Supplementary Figure 5). A statistically inverse correlation (*P* < 0.05, Spearman's test) between serum adalimumab levels and clinical laboratory variables, including disease activity score, CRP, and fecal calprotectin was observed (Supplementary Figure 6). The adalimumab cutoff value for sustained remission (i.e., 13.85 μg/ml at the end of induction) significantly correlated with fecal calprotectin measured during the induction: patients not achieving the cutoff for adalimumab concentrations had significantly higher fecal calprotectin compared with others, and marginally significant results were observed for CRP (Supplementary Figure 7).

### Correlation Between ADA Levels and Drug Concentration

ADA concentrations were measured in 9 samples from 6 patients (18.8%) that showed serum adalimumab levels below 1.5 μg/ml. Only 4 samples from 2 patients (6.3%) resulted positive to ADAs. None had previously developed anti-infliximab antibodies. ADA concentrations were inversely correlated with adalimumab levels, but the correlation was marginally significant (Spearman test, *P* = 0.073).

## Discussion

This study confirmed that serum adalimumab levels correlate with clinical remission and showed that higher adalimumab levels in the first weeks of treatment predict a long-term remission. Most of the patients were treated with adalimumab after infliximab, and the overall efficacy is comparable with previous reports (19).

The adalimumab concentration's cutoff value of 13.85 μg/ml at week 4 was found to have a PPV above 83.3% in reaching remission at weeks 52 and 82 in children with IBD, while an adalimumab concentration's cutoff of 7.54 μg/ml was observed when serum adalimumab levels were measured at week 22. These findings are in line with studies in adult patients, which showed that clinical remission and mucosal healing after 52 weeks of treatment could be predicted from adalimumab trough levels above 5 μg/ml measured at week 26 (13, 20, 21). Similarly, it has been recently reported that in children with CD that have clinically responded to the first two doses of adalimumab, maintaining trough levels above 5 μg/ml starting on week 4 with a proactive strategy is associated with a remission rate of 82% at week 72 (22). It could be hypothesized that adalimumab dose could be intensified using drug concentrations measured at a definite time point (end of induction) to improve long-term efficacy. In this context, Rinawi and colleagues have found that a higher induction dose in the early phase of therapy correlates positively with adalimumab trough levels at weeks 4 and 8 (22.5 and 12.5 μg/ml, respectively), leading to clinical remission at week 24, in pediatric IBD patients with CD (23). Clinical utility of the pharmacological testing in adalimumab-treated patients even in the absence of trough levels has already been proved in other diseases (24). In a cohort of adults with rheumatoid arthritis, low adalimumab levels and ADAs at 3 months, not obtained at trough but during routine clinical visits, were significant predictors of no response after 1 year of treatment (25). In clinical routine, it may not always be possible to obtain serum just before the patient is due to receive the next injection. Therefore, studies in the context of drug measured at non-trough are essential. Adalimumab administration is advantageous because of patient self-injection at home; the drug is indeed administered through subcutaneous injections, and this allows for slower absorption and distribution than infliximab, which is delivered via intravenous infusions. Besides, the administration of adalimumab is performed at closer intervals than infliximab, and its half-life is longer than infliximab (26). For these reasons, the use of adalimumab non-trough levels may be reliable (24). Interestingly, particularly in responders, adalimumab concentration was higher when measured at 4 weeks than at 22 weeks. This difference is not determined by a different dose; therefore, other reasons should be considered and could involve production of ADAs, differences in TNF-alpha concentration that may lead to a higher adalimumab clearance (27), or differences in disease location that may lead to drug loss through the intestine (28). More studies are required to confirm this observation and evaluate the mechanisms and clinical implications.

This study confirmed an inverse correlation between drug levels and biochemical variables, in particular, CRP and fecal calprotectin (29) and the absence of a significant effect on adalimumab concentration of concomitant therapy with immunosuppressants, as previously reported (30). ADAs developed in 6.3% of the patients, and a marginal inverse correlation was found between drug and antibody concentration. Previous studies showed that the ADA incidence is between 0.04 and 35% (31). In the IMAgINE pediatric study, 3.3% of the patients developed ADAs, while in the PAILOT study 10% of patients and their levels correlated with low drug levels, similar to what was observed in our study (22). A previous study conducted by our group demonstrated that 20% of the patients were positive to anti-infliximab antibodies. Therefore, in this cohort, adalimumab appears less immunogenic than infliximab (6 vs. 20% of patients have developed ADAs) (10).

## Limitations

This study has several limits starting from its retrospective design that may affect the homogeneity of the data and the relatively small size of the cohort that prevents adjusting the analysis for different indications of treatment in the CD and UC subgroups. About 30% of the patients started adalimumab in clinical remission, although with laboratory signs of clinically active disease. This may have influenced the number of patients in remission at the various time points but not the correlation between drug levels and the short- and long-term outcomes, which was the aim of this study. Moreover, the use of non-trough adalimumab levels limits the direct comparison with previous adult and pediatric studies on TDM, which evaluated adalimumab levels at trough. Endoscopic evaluation was not performed as part of the study design, and data on mucosal healing were not available. Still, fecal calprotectin values were collected as a good surrogate of mucosal healing (32). Finally, due to the technical characteristics of the ELISA assay, which does not permit to measure ADAs in the presence of adalimumab (above 1.5 μg/ml), we have analyzed the levels of ADA only in six patients, thus limiting to draw any conclusion between adalimumab levels and ADAs.

## Conclusion

In conclusion, adalimumab levels, ascertained from non-trough level serum samples, are associated with long-term response in pediatric patients with IBD. Further prospective studies evaluating the use of non-trough levels in the proactive algorithm or the use of a single time point, for instance, end of induction, to predict long-term response are needed.

## Data Availability Statement

The raw data supporting the conclusions of this article will be made available by the authors, without undue reservation.

## Ethics Statement

The studies involving human participants were reviewed and approved by Comitato Etico Unico Regionale - CEUR. Written informed consent to participate in this study was provided by the participants' legal guardian/next of kin.

## Author Contributions

GS and MB conceptualized and designed the study. ML, DC, and GS acquired, analyzed, and interpreted the data, and drafted the initial manuscript. PA, SM, TS, VG, and FL acquired the data. GD critically discussed and supervised the study. All authors contributed to the article and approved the submitted version.

## Conflict of Interest

The authors declare that the research was conducted in the absence of any commercial or financial relationships that could be construed as a potential conflict of interest.
