# Supplementary material for: Serum Adalimumab Levels After Induction Are Associated With Long-Term Remission in Children With Inflammatory Bowel Disease
Source: Front Pediatr. 2021 May 4;9:646671. doi: 10.3389/fped.2021.646671 (PMC8129012; doi:10.3389/fped.2021.646671)
Supplement: Supplementary file 1 [file Data_Sheet_1.PDF]

Supplementary Material

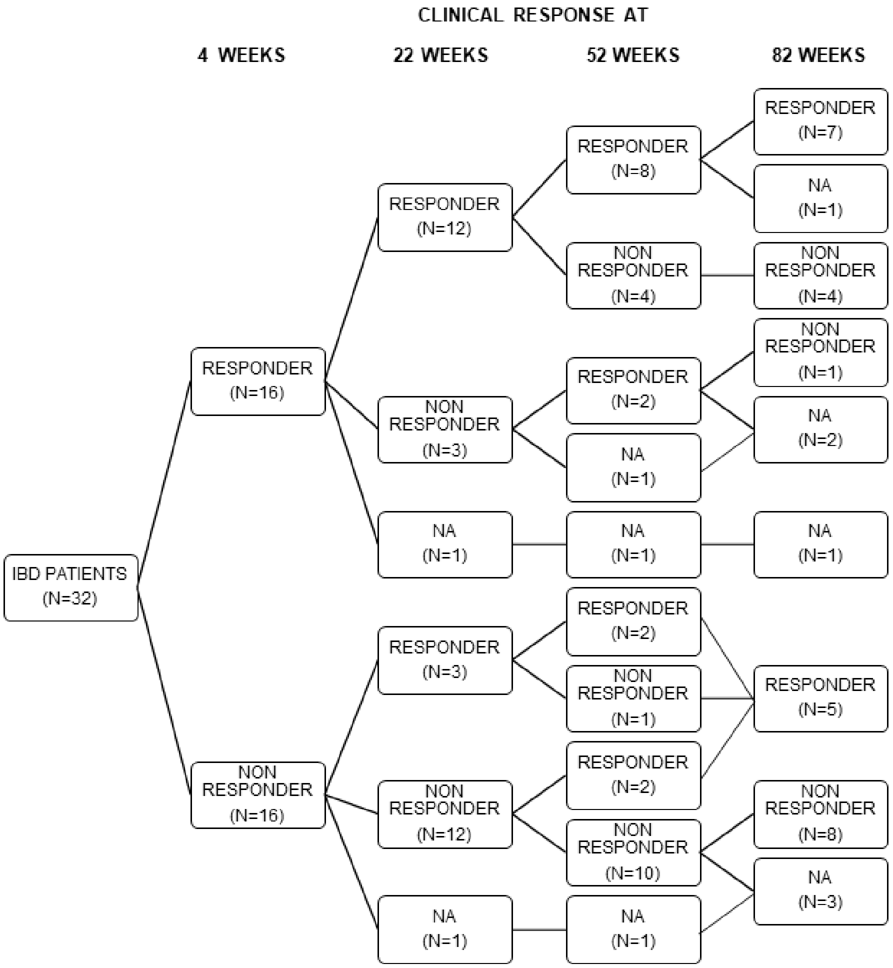

Supplementary Figure 1. Flow chart of IBD patients enrolled in the study. Responders and non-responders to adalimumab therapy were assessed at defined time points (at weeks 4, 22, 52 and 82).

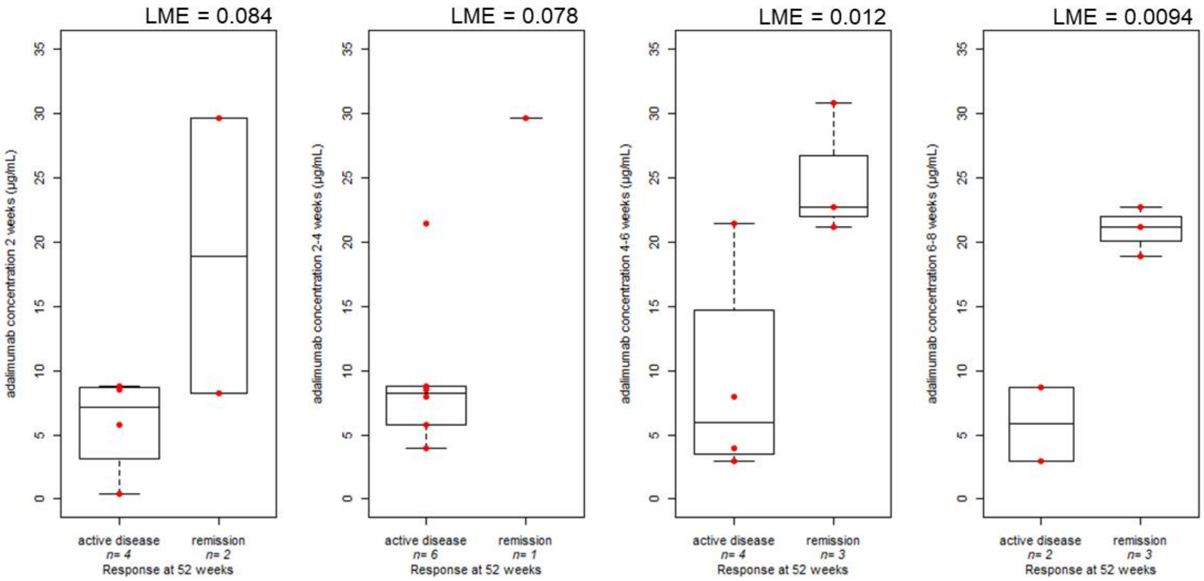

Supplementary Figure 2. Correlation between long term response and adalimumab levels considering shorter timeframes in the 2-8 weeks interval.

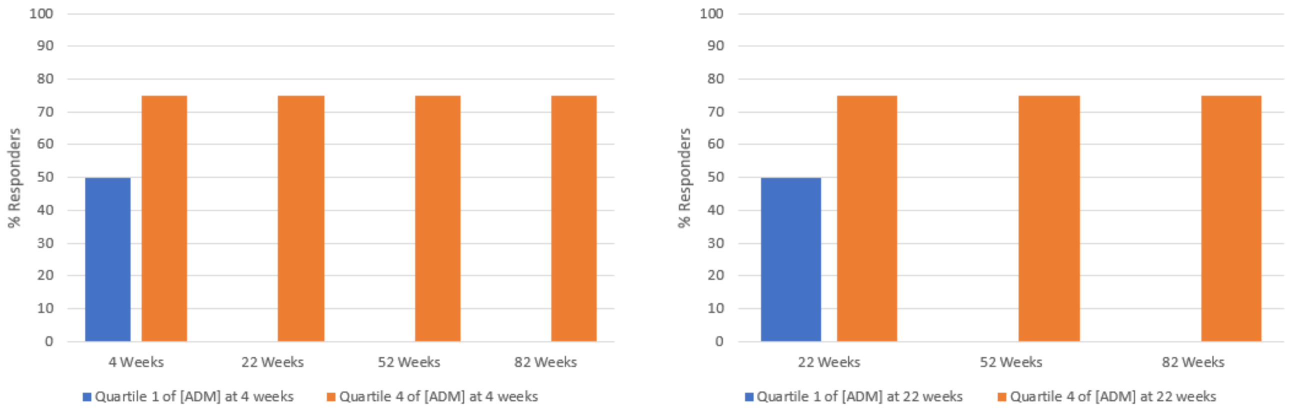

Supplementary Figure 3. Quartile analysis (Quartile 1, low exposure vs Quartile 4, high exposure) of adalimumab concentrations and clinical response at different time points.

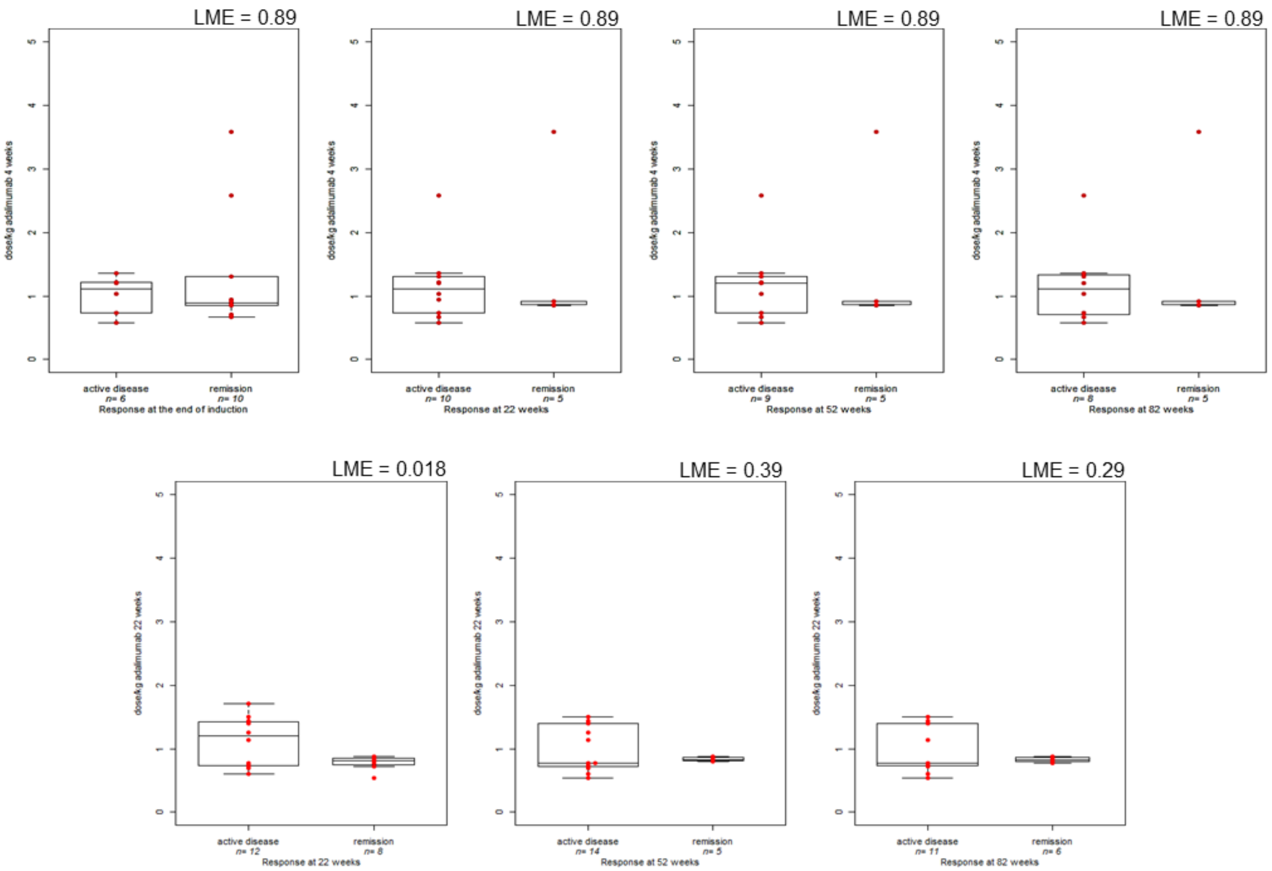

Supplementary Figure 4. Correlation between clinical response and adalimumab levels after dose adjustment normalised on patients' weight.

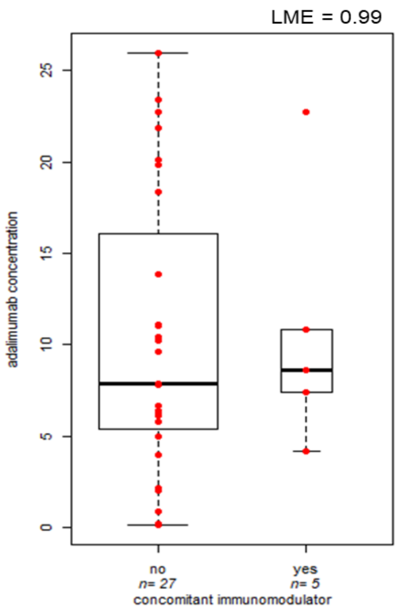

Supplementary Figure 5. Correlation between adalimumab levels and concomitant treatment with immunomodulators.

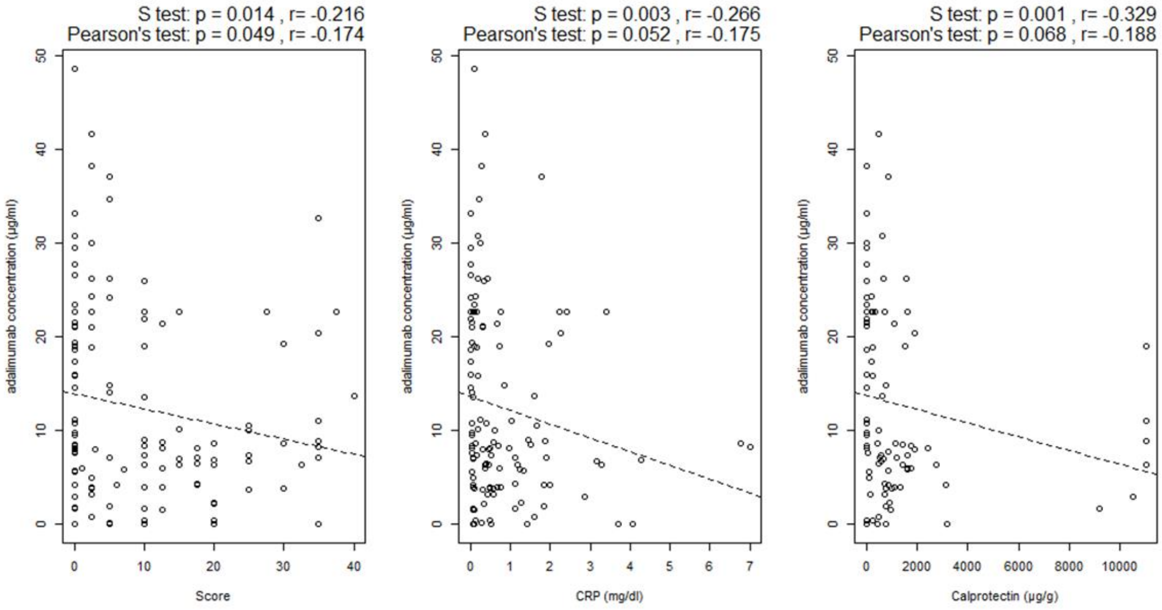

Supplementary Figure 6. Concentration of adalimumab was significantly inversely correlated with the clinical score (129 measurements in 32 patients), CRP (124 measurements in 32 patients) and fecal calprotectin (95 measurements in 27 patients).

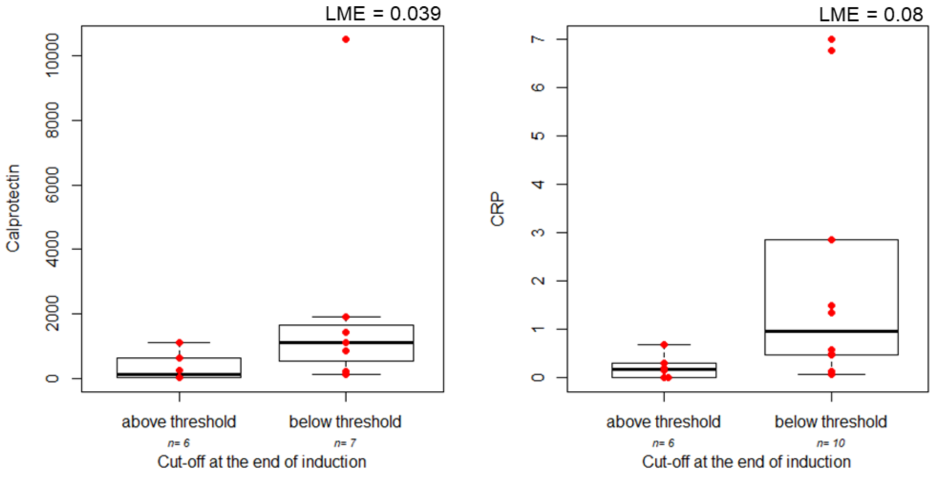

Supplementary Figure 7. Levels of CRP and calprotectin in patients with a concentration of adalimumab below or above the threshold level associated with sustained response.

Table 1S. Optimal cut-off values at week 4 and 22 to predict long-term response.

	**Cut-off at the end of induction (µg/mL)**	**Responder/Non responder**	**Odds Ratio [Confidence Interval]**	**Logistic regression (p-value)**
**Response At 52 weeks**	≥ 13.85<br>< 13.85	5 / 1<br>1 / 8	40 [2-794.3]	0.015
**Response at 82 weeks**	≥ 13.85<br>< 13.85	5 / 1<br>0 / 8	62.33 [2.13 – 1822.7]	0.016

	**Cut-off at 22 weeks (µg/mL)**	**Responder/Non responder**	**Odds Ratio [Confidence Interval]**	**Logistic regression (p-value)**
**Response At 52 weeks**	≥ 7.54<br>< 7.54	5 / 3<br>1 / 12	20 [1.65-241.7]	0.018
**Response at 82 weeks**	≥ 10.51<br>< 10.51	4 / 1<br>3 / 11	14.66 [1.16-185.24]	0.037
